# Monitoring and Ensuring Worker Health in Controlled Environments Using Economical Particle Sensors

**DOI:** 10.3390/s24165267

**Published:** 2024-08-14

**Authors:** Juan Antonio Rodríguez Rama, Leticia Presa Madrigal, Jorge L. Costafreda Mustelier, Ana García Laso, Javier Maroto Lorenzo, Domingo A. Martín Sánchez

**Affiliations:** 1 Escuela Técnica Superior de Ingenieros de Minas y Energía, Universidad Politécnica de Madrid, C/Ríos Rosas, 21, 28003 Madrid, Spain; leticia.presa.madrigal@upm.es (L.P.M.); jorgeluis.costafreda@upm.es (J.L.C.M.); ana.garcia.laso@gmail.com (A.G.L.); javier.maroto@upm.es (J.M.L.); domingoalfonso.martin@upm.es (D.A.M.S.); 2 Laboratorio Oficial para Ensayos de Materiales de Construcción (LOEMCO), C/Eric Kandell, 1, 28906 Getafe, Spain

**Keywords:** monitoring, health, air quality, particulate matter, low-cost sensors

## Abstract

Nowadays, indoor air quality monitoring has become an issue of great importance, especially in industrial spaces and laboratories where materials are handled that may release particles into the air that are harmful to health. This study focuses on the monitoring of air quality and particle concentration using low-cost sensors (LCSs). To carry out this work, particulate matter (PM) monitoring sensors were used, in controlled conditions, specifically focusing on particle classifications with PM2.5 and PM10 diameters: the Nova SDS011, the Sensirion SEN54, the DFRobot SEN0460, and the Sensirion SPS30, for which an adapted environmental chamber was built, and gaged using the Temtop M2000 2nd as a reference sensor (SRef). The main objective was to preliminarily assess the performance of the sensors, to select the most suitable ones for future research and their possible use in different work environments. The monitoring of PM2.5 and PM10 particles is essential to ensure the health of workers and avoid possible illnesses. This study is based on the comparison of the selected LCS with the SRef and the results of the comparison based on statistics. The results showed variations in the precision and accuracy of the LCS as opposed to the SRef. Additionally, it was found that the Sensirion SEN54 was the most suitable and valuable tool to be used to maintain a safe working environment and would contribute significantly to the protection of the workers’ health.

## 1. Introduction

Air pollution is one of the greatest environmental threats to human health, together with climate change. This has led to the lowering of air quality reference level limits by both the World Health Organization (WHO) and individual governments [1,2,3,4,5]. By not exceeding these reference levels, countries protect public health and contribute to mitigating global climate change [6,7].

Air quality is an essential component of public health, especially in industrial settings where activities such as the production of cement, ceramics, and gypsum generate particulate matter [8,9]. This material is generally classified by size into PM2.5 and PM10 fractions, considered crucial indicators in air quality indexes due to their significant impact on health [10,11]. Particles vary not only in size but also in composition and origin, factors that directly influence their effects on human health [12].

Industrial origin particles, resulting from processes such as the combustion of fossil fuels and the manufacturing of building materials, often contain heavy metals and other harmful chemical compounds. Meanwhile, particles from natural sources, such as desert dust and marine aerosols, although generally less toxic, can also adversely affect respiratory and cardiovascular health [13].

Prolonged exposure to PM2.5 and PM10 is associated with various chronic health issues, with PM2.5 particles revealing more severe health effects as they can penetrate deeply into the respiratory system to the pulmonary alveoli and enter the bloodstream, increasing the likelihood of producing toxic effects in the recipient. In the workplace, inhaling these particles can lead to occupational diseases such as pneumoconiosis, a group of lung conditions caused by the accumulation of dust in the lungs, which trigger a significant reaction in the lung tissue due to the deposited particles. Silicosis is one of the most common forms of pneumoconiosis. Additionally, finer and ultrafine particles have the capacity to penetrate deeply into the respiratory tract and circulatory system, exacerbating or triggering chronic conditions such as asthma and bronchitis, and increasing the risk of serious cardiovascular events, such as heart attacks and strokes [14,15,16,17].

Control and monitoring of these particles are essential to reduce the exposure of the population to these pollutants [18]. Traditional monitoring methods are based on 24 h particle collection on filters or satellite surveys [19,20,21], which require expensive equipment, skilled personnel, and complex calibration procedures. In addition, air pollution varies spatially and temporally, requiring large and dense monitoring networks to adequately detect pollution peaks [22,23]. Furthermore, extrapolating these systems to indoor monitoring of spaces is complicated by their size and the need for rapid warnings in highly exposed environments [24,25].

Over the last decade, there has been a considerable increase in interest in developing portable and economical sensors to measure PM2.5 concentrations, due to needs in research and occupational health. These devices rely on optical methods that, despite their speed, have limitations in detecting particles smaller than 300 nm and are susceptible to changes in particle size, shape, and composition. Variations in environmental humidity can also negatively affect the accuracy of the measurements [26,27].

Recent advancements in low-cost sensor technologies have opened new possibilities for monitoring particulate matter in indoor environments. These sensors are compact, affordable, and capable of transmitting large volumes of data in real time to facilitate rapid analysis. However, it is crucial that their accuracy, reliability, and factory calibrations be rigorously evaluated and validated to ensure the reliability of the data obtained. Although they offer practical advantages and new monitoring opportunities, these devices still lack some functionalities of regulatory measurement equipment, which requires careful analysis of the reliability of their measurements [28,29,30].

The Laboratorio Oficial para Ensayos de Materiales de Construcción (LOEMCO) [31] is integrating digital particle monitoring technologies in critical areas of work. In collaboration with Tellus UPM [32], the Social Entrepreneurialism, Ethics and Values in Engineering (UESEVI) from the Escuela Técnica Superior Ingenieros de Minas y Energía of the Universidad Politécnica de Madrid (ETSIME-UPM) has developed an ecosystem that integrates Industry 4.0 technologies such as the Internet of Things (IoT) [33], Big Data, 3D printing [34], and data-driven culture. This joint effort seeks to improve the continuous monitoring and environmental management of indoor spaces, increasing efficiency, sustainability, and ensuring the safety and health of personnel.

The main objective of this preliminary study is to investigate the accuracy and reliability of LCSs for measuring PM2.5 and PM10, comparing them with reference methods in a controlled environment. This approach will evaluate the feasibility of using these devices in monitoring networks. The results of this research could serve as a basis for future research to validate the routine use of LCSs for monitoring environmental parameters, necessary for the development of an indoor monitoring network.

## 2. Materials and Methods

### 2.1. Materials

**Figure 1:**
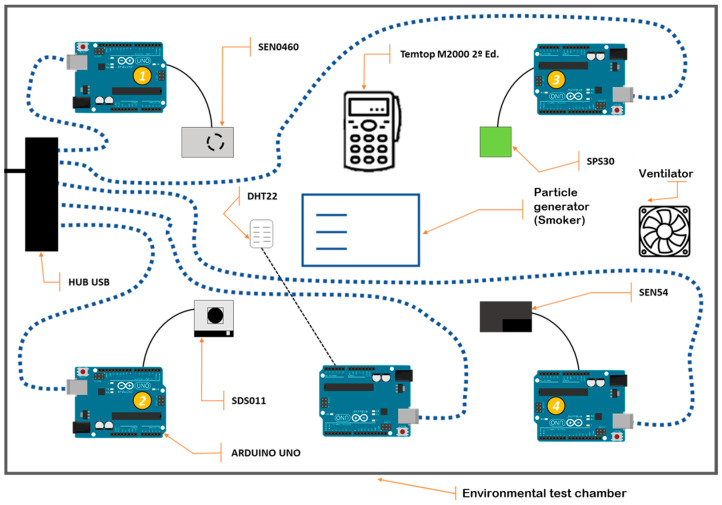
Schematic of experimental design for LCS analysis.

The study was conducted in an environmental simulation chamber; the schematic is shown in Figure 1 with temperature and humidity values within the operating parameters of the LCS and SRef. These conditions, which affect the measurement of particulate matter, were kept under control to validate the sensors, which had negligible fluctuations throughout the test.

A fan in the chamber ensures uniform particle distribution, simulating ambient conditions and avoiding stratification. The LCSs and SRef are arranged in a rectangular pattern to minimize the edge effect and ensure the validity of the experiment. This provides a replicable test environment, minimizes measurement biases, and maintains data integrity, ensuring robust and replicable conclusions for the evaluation of sensor technologies in PM2.5 and PM10 monitoring.

The chamber’s dimensions are 50 × 70 × 50 cm and it simulates environmental conditions on a reduced scale. Its robust structure is due to the 0.5 cm methacrylate and reinforcements designed with Autodesk’s Fusion 360 software (https://www.autodesk.com/es/products/fusion-360/overview?term=1-YEAR&tab=subscription, accessed on 1 August 2024) and printed in 3D using Ultimaker’s Cura software (v. 5.7.1., https://ultimaker.com/software/ultimaker-cura/, accessed on 1 August 2024) and a Creality Ender 3 3D printer (Shenzhen, China) using standard PLA. The transparency of the material allows visual observation, the top lid facilitates internal manipulation, and the seal prevents air leakage to alter internal concentrations.

A smoker using wood chips and paper as combustion agent was selected as a source of particulate matter (PM2.5 and PM10) to evaluate the capacity of the sensors [35]. This device simulates real situations, such as forest fires, and provides a platform to analyze the response of the sensors. The slow and homogeneous ignition improves the evaluation of LCSs. Its use guarantees a constant generation of particulate matter, simulating a common source of pollution in urban and industrial environments. This configuration provides a robust platform for evaluating the effectiveness and accuracy of LCSs compared to SRef.

Among a wide variety of commercially available devices, the technical characteristics of nine of these devices were analyzed (the Sensirion SEN54, the Sensirion SPS30 (Sensirion, Zurich, Switzerland), the Nova SDS011 (Nova Fitness Co., Jinan, China), the TeraSensor Next-PM (Groupe Tera, Rousset, France), the DFRobot SEN0460 (DFRobot, Chengdu, China), the BJHIKE HK-A5 (Bjhike, Beijing, China), the Samyoung DSM501 (Samyoung S&C, Seongnam, South Korea), the Panasonic SN-GCJA5 (Panasonic Photo & Lighting Co., Osaka, Japan), and the Sharp GP2Y10 (SHARP, Osaka, Japan)), from which four LCSs were selected. These were the Sensirion SEN54 and SPS30, which use optical technology to detect particles with a measurement range of 0 to 1000 µg/m^3^ and an accuracy of ±10 µg/m^3^ + 15%. They are compatible with UART and I2C interfaces, comply with international safety and environmental standards, and feature a self-cleaning system.

The Nova SDS011 uses laser technology to measure particle concentrations between 0 and 999.9 µg/m^3^ with an accuracy of ±10 µg/m^3^ or ±15% and it stands out for its fast response and low cost. And finally, the DFRobot SEN0460, noted for its compact design and energy efficiency, can measure particles with an accuracy of ±10% in ranges from 100 to 500 µg/m^3^ and operate in a temperature range of −10 to 60 °C with humidity up to 95%.

The Temtop M2000 2nd (Temtop, San Jose, CA, USA) is used as a portable, multifunctional air quality detector for comparative testing. This instrument provides accurate measurements of various pollutants, including PM2.5, PM10, CO_2_, and formaldehyde, using laser and NDIR sensor technology [36,37]. With a measurement range of 0–999 µg/m^3^, a resolution of 0.1 µg/m^3^, and an accuracy for PM2.5 of ±10 µg/m^3^ (0–100 µg/m^3^); ±10% (100–500 µg/m^3^) and ±15 µg/m^3^ (0–100 µg/m^3^); ±15% (100–500 µg/m^3^) for PM10, it allows detailed air quality monitoring. The AQ-SPEC evaluation [38] proved to be consistently accurate with 55% to 70% and a strong correlation with FEM reference instruments. Despite weaker correlations in PM10 measurements, it remains useful for assessing air quality where a reliable reference standard for PM2.5 is required. Its robust characteristics, low intramodel variability, and 100% data recall ensure its reliability in diverse environments and its usefulness for LCS comparison and evaluation.

The Arduino UNO microcontroller (Turin, Italy), selected for the PM10 and PM2.5 particulate matter monitoring test, stands out for its compatibility, flexibility, and cost-effectiveness. Its open hardware platform and extensive software library facilitate multiple sensor integration and data comparison. Arduino’s integrated development environment (IDE) is accessible, optimizing time and resources. The user community and technical support provide additional support during project development.

The data storage capacity of the microcontroller, through external modules such as SD cards, allows long-term data accumulation. Compatible microSD shields provide significant capacity in a compact form factor; they are also affordable and allow easy data transfer. Their reliability ensures data preservation in the event of power outages. These attributes make the use of microSD shields an optimal solution for data storage in air quality monitoring projects.

### 2.2. Methods

For the test, it was assumed that the selected LCSs were adequately precalibrated and factory validated, as indicated in their data sheets [39,40,41,42], to guarantee accuracy and reliability in the measurements of particulate matter, specifically PM2.5 and PM10. Consequently, a continuous measurement protocol was implemented, recording data every five minutes on the microSD cards.

**Figure 2:**
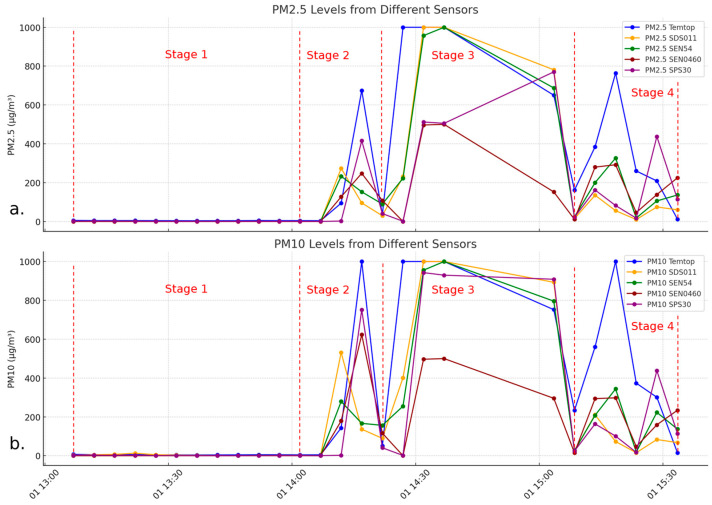
Overview of the data obtained throughout the test with PM2.5 (**a**) and PM10 (**b**).

The experiment consisted of four phases. In the first phase, ambient baseline levels were recorded, observing near-zero particle levels with the urn open, as shown in Figure 2a,b. In the second phase, with the urn closed, a specific contribution of particles was generated, with sharp increases and decreases in concentration. In the third phase, also with the urn closed, the SRef was saturated with PM2.5 and PM10 values of 999 µg/m^3^, with a progressive decrease in concentration. Finally, in the fourth phase, concentration stages were set with progressive increases and decreases.

During phases 2, 3, and 4, the fan was switched on to ensure homogeneous particle distribution. This process evaluates the response of the sensors to different levels of particle concentration and their behavior under changes in ventilation and air access.

#### 2.2.1. Validation by Statistical Techniques

The statistical validation procedure to evaluate the accuracy and reliability of the sensors incorporates robust techniques, ensuring objectivity and thoroughness. During the test, PM2.5 and PM10 data were collected for each LCS and aggregated into a file to enable detailed analysis.

Initial review of the data identifies trends and outliers. Matrix plots, box plots, and linear regression analysis allowed us to assess the relationships between variables by providing a graph of the data distribution, allowing us to quickly identify the median, quartiles, and outliers. Pearson and Spearman correlations were used to evaluate the linear and monotonic relationships between the data.

To quantify the average error in the predictions, the mean absolute error (MAE) and root mean squared error (RMSE) are used. Acceptable values for these indicators depend on the specific context of the analysis, but, generally, lower MAE and RMSE indicate better predictions.

Lin’s concordance coefficient (CCLin) evaluates the agreement between two measurement methods. A CCLin close to 1 indicates high correlation.

Student’s t-distribution compares the means of two groups. A *p*-value less than 0.05 generally indicates a significant difference between the means.

To perform these statistical calculations, the Python programming language was used, along with some of its libraries, such as Pandas, Numpy, Matplotlib, SciPy, and Scikit learn, that add functionalities that allowed us to evaluate the accuracy and reliability of the LCS compared to the SRef. This section details the results obtained from the analysis.

These methods and thresholds guarantee a detailed evaluation of the accuracy and reliability of the sensors [43,44,45], thus allowing a rigorous and objective validation of the sensors.

#### 2.2.2. Monitoring by Thermographic Imaging

This technique allows real-time visualization of the temperature distribution in the test chamber, which can influence the dynamics of PM2.5 and PM10, for which a calibrated E60bx thermographic camera from Testo (Titisee-Neustadt, Germany) was used. These images help to identify unusual and anomalous readings.

For thermal stability monitoring, a controlled test environment was maintained, fluctuations requiring adjustments were identified, and the reliability and accuracy of the data obtained were ensured.

## 3. Results

The results obtained from the study of the LCS compared to the SRef in a controlled environment are presented below. This analysis was carried out to evaluate the accuracy and reliability of the LCSs in the measurement of PM2.5 and PM10 particles, with the objective of providing a detailed view of the performance of each sensor under different experimental conditions.

Figure 2a,b show the final results of the data obtained from applying the methodology described in Section 2.2.

### 3.1. Box Plot Data

**Figure 3:**
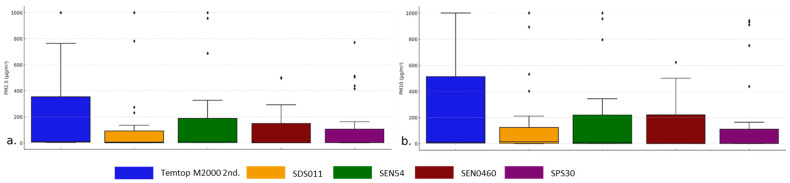
Box plot of PM2.5 (**a**) and PM10 (**b**) measurements from different sensors.

For PM2.5 readings, the SRef recorded moderately high levels, as shown in Figure 3a, and depicts few extreme values, indicating a stable measurement. SDS011 exhibits a wide distribution and greater variability in measurements, including some high outliers. This indicates higher sensitivity, from factory calibration or exposure to PM2.5 sources. SEN54 and SEN0460 show the lowest distributions, indicating comparatively lower measurements. SEN54, on the other hand, includes some outliers, which requires attention in its calibration or location. Finally, SPS30 shows intermediate variables, with a more centered distribution than SDS011, but without extremes, implying more consistent and controlled measurements.

For PM10 readings, the Temtop M2000 2nd behaves similarly to that of PM2.5, as shown in Figure 3b, where high PM10 levels are seen with some extreme values, indicating a possible higher exposure to large particles. SDS011 suggests high variability and the highest values among the sensors, which also proves higher sensitivity or exposure to large particles. Finally, the SEN54, SEN0460, and SPS30 sensors have more stable distributions, indicating less variability in their PM10 measurements. This reflects greater accuracy and control, as well as better calibration of these devices. In addition, it shows high PM10 levels with some extreme values, indicating a possible higher exposure to large particles.

SDS011 shows high variability and the highest values among the sensors, which also confirms a higher sensitivity or exposure to large particles. Finally, the SEN54, SEN0460, and SPS30 sensors have more stable distributions, indicating lower variability in their PM10 measurements, reflecting higher accuracy and control, and indicating better factory calibration of these devices (Figure 3b).

### 3.2. Matrix Plots of the Data

**Figure 4:**
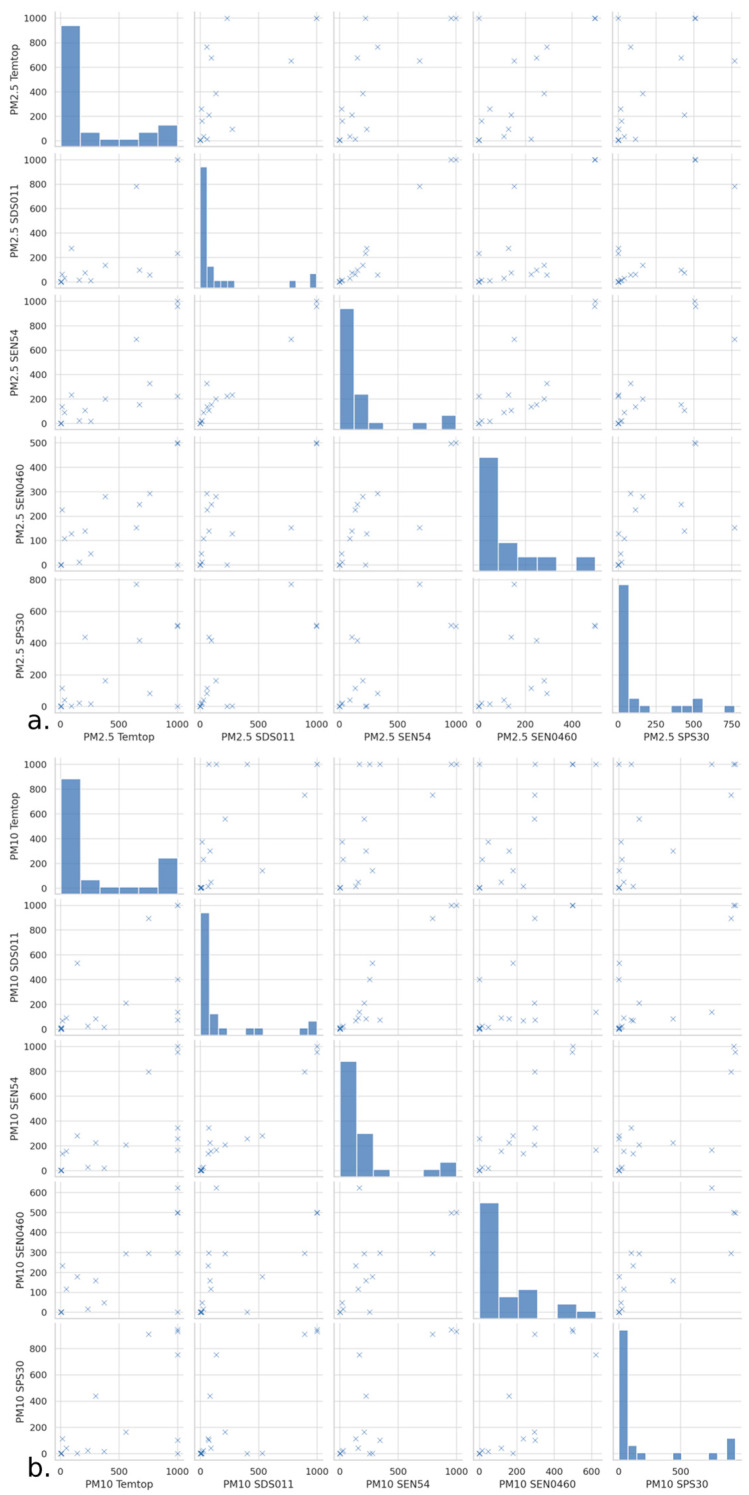
Matrix plots (pair plots) for PM2.5 (**a**) and PM10 (**b**) measurements from different sensors.

The PM2.5 plot shows (Figure 4a) a positive correlation between the sensors, specifically between SRef and SDS011, indicating a tendency to record similar PM2.5 levels under similar conditions.

The distribution of PM2.5 varies among the sensors, as can be seen in Figure 4b. Some sensors, such as SDS011, show a wider dispersion, while others, such as SEN54 and SEN0460, show more centered and compact distributions. This could be due to differences in sensor sensitivity or calibration.

The similarity in behavior between PM2.5 and PM10 readings from the LCSs can be seen in Figure 4b. Similar behavior to that observed with PM2.5 is observed, showing a positive correlation between their measurements, although some correlate less with other sensors, as is the case of SEN0460, which could be indicative of differences in the ability to detect larger particles. PM10 distributions also vary with sensors such as the SDS011, showing wider ranges of measurements, which may indicate greater exposure or sensitivity to PM10 size particles.

### 3.3. Linear Regression Analysis and Coefficient of Determination (R^2^) of the Data

**Figure 5:**
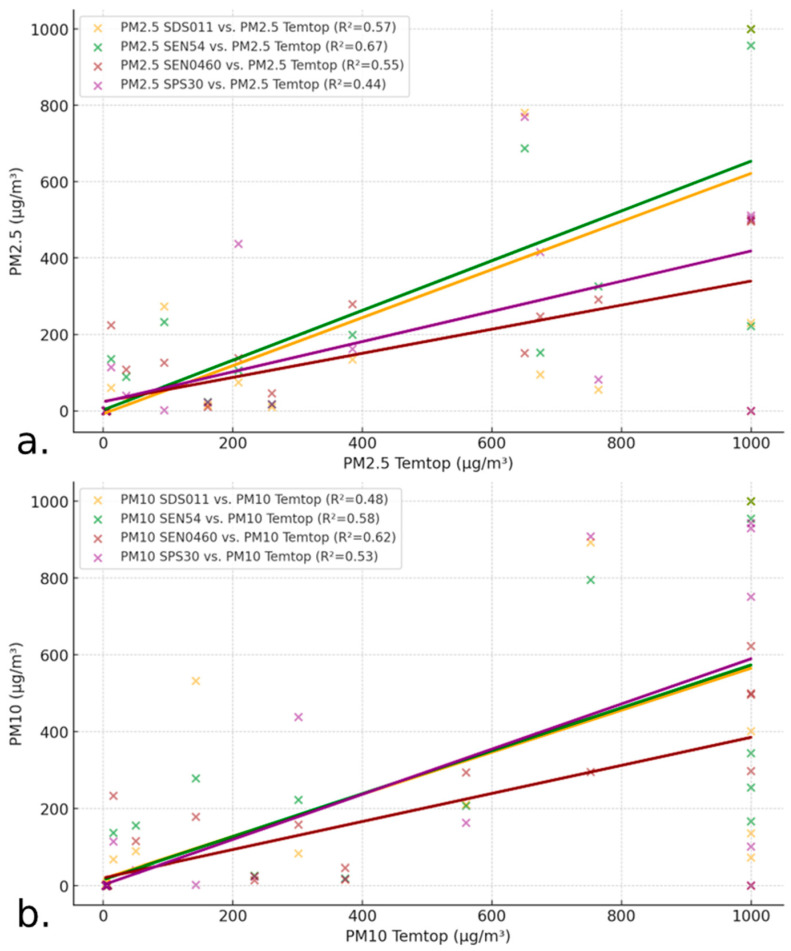
Matrix plots (pair plots) for PM2.5 (**a**) and PM10 (**b**) measurements of the different sensors.

To compare how the LCS measurements are related to the SRef, the PM2.5 data were analyzed, and we observed that the SDS011 and SEN54 sensors had a better correlation with the SRef than the SPS30 and SEN0460 (Figure 5a), indicating higher accuracy or similar sensitivity to environmental conditions.

On the other hand, the R^2^ values for PM10 and PM2.5 show a moderate correlation, with values between 0.30 and 0.50, indicating that the sensors show similar trends to those of the SRef, but with a variability in the accuracy (Figure 5b). In general, the correlation for PM10 is like that of PM2.5, with SEN54 and SEN0460 showing the best overall fit.

### 3.4. Correlation of the Data According to the Pearson and Spearman Matrix

**Figure 6:**
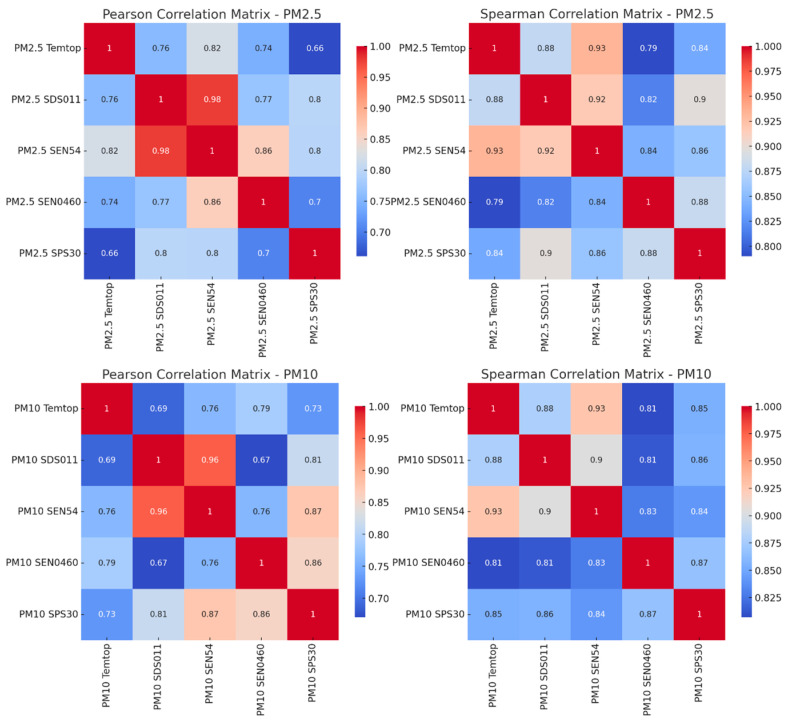
Pearson and Spearman correlation matrix for PM2.5 and PM10.

The results of both coefficients (Figure 6) are consistent, reinforcing the validity of the LCS measurements compared to the SRef.

For PM2.5, the coefficients calculated by Pearson’s correlation show moderate to high values between SRef and LCS, indicating good linearity. At the same time, those calculated by Spearman also show moderate to high correlations, suggesting that the ranges of measurements are aligned between sensors.

For PM10, we observed moderate to high values in the Pearson coefficients, indicating that the PM10 measurements of the LCSs rise and fall in accordance with the SRef. Those calculated by Spearman correlation are high, indicating a strong relationship in terms of rankings and trends, although not linear.

The high correlations show that although there may be lower precision and variability among LCSs, they generally follow SRef trends.

The variability and differences in coefficients underscore the need for careful calibration and validation of each sensor prior to use in critical applications.

### 3.5. Mean Absolute Error (MAE) and Root Mean Squared Error (RMSE) of the Data

The MAE and RMSE values (Table 1) show the accuracy and variability of each sensor compared to the SRef. Generally, the errors are considerable, indicating that, although the LCSs may follow similar trends as the SRef, their accuracy is limited to the factory calibration used in their evaluation.

SEN54 shows better performance in terms of MAE and RMSE compared to the other sensors, especially at PM2.5, indicating greater data consistency and reliability. The highest values of errors in PM2.5 and PM10 are found in SEN0460, which could reflect calibration problems.

### 3.6. Lin’s Concordance Coefficient, Lin’s Concordance Correlation Coefficient (CCLin or CCC) of the Data

The overall agreement shows that the Lin’s coefficient of concordance (CCLin) values reflect good alignment of the LCS measurements with the SRef, as can be seen in Table 1.

For PM2.5 values, SEN54 has the best CCLin (0.774), showing good alignment with the SRef. SDS011 (0.712) also shows good concordance, while SEN0460 and SPS30 (0.471 and 0.534, respectively) have lower values, indicating lower concordance and possibly higher variability in their measurements.

Considering the PM10 values, SPS30 and SEN54 (0.675 and 0.689, respectively) show the best agreement, indicating a good correlation with the SRef measurements. Sensors SDS011 and SEN0460 show a moderate agreement, with CCLin values of 0.640 and 0.526, respectively.

### 3.7. Student’s t-Distribution of the Data

As can be seen in Table 1, for both PM2.5 and PM10, none of the LCSs show statistically significant differences compared to SRef. This is because all *p*-values are greater than 0.05. Although the SEN0460 sensor shows the highest F-value for both measurements, with *p*-values close to the threshold of significance (0.07 and 0.067 for PM2.5 and PM10, respectively), it suggests that this sensor could portray a significant difference to the other sensors. These results would indicate that, in terms of consistency with the SRef, the LCSs perform similarly to each other.

## 4. Discussion

**Figure 7:**
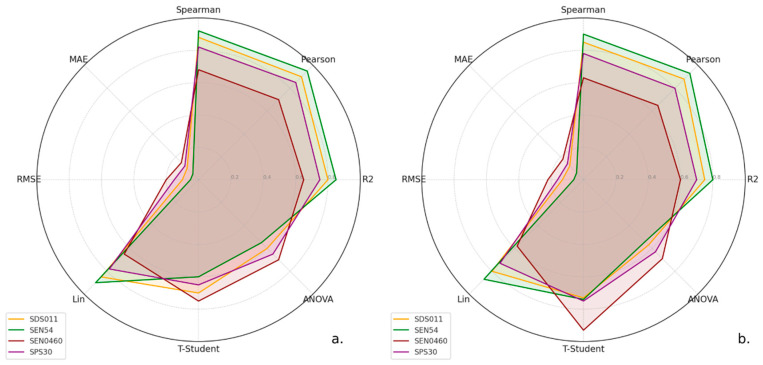
Radar plots synthesizing the performance of multiple LCSs vs. SRef. (**a**) Radar plot for PM2.5 values and (**b**) radar plot for PM10 values.

For the discussion of the statistical analysis in this study, radar plots for PM2.5 and PM10 values are used (Figure 7a and Figure 7b, respectively). These plots allow us to synthesize the various statistical behaviors and evaluate the effectiveness of the LCS compared to the SRef. Values close to zero for mean absolute error (MAE) and root mean squared error (RMSE) are considered good indicators of performance, signaling higher accuracy. For Lin’s coefficient of concordance (CCLin or CCC), values close to 1 denote excellent agreement between two measurement methods. For Pearson’s and Spearman’s correlation coefficients, values close to +1 or −1 reflect a strong correlation, either linear or monotonic, indicating alignment in the data ranges. In the analysis of variance (ANOVA) and Student’s *t*-test, a low *p*-value (less than 0.05) suggests statistically significant differences between groups. The coefficient of determination (R^2^) measures the proportion of variability in the dependent variable that can be explained by the independent variables. High values, close to 1, are ideal and show that the model captures a large part of the observed variability.

As seen in Figure 7a for PM2.5, SDS011 and SEN54 show robust performance on most metrics, especially Pearson and Spearman, indicating strong correlations. The SEN0460 and SPS30 sensors have lower performances on several metrics, especially on concordance measures and significance tests. SEN54 tends to have lower errors (MAE and RMSE values), showing more accurate performance. The inverted Student’s t and ANOVA values suggest that, in general, there are no significant differences in means for most sensors. This would agree with similar projects [46,47].

The radar plot for PM10 (Figure 7b) shows that SDS011 and SEN54 stand out for their correlations, while SEN0460 shows a lower performance. SPS30 shows an intermediate performance. On the other hand, SEN54 continues to show lower errors, and no significant differences in the measurements are observed for most of the LCSs.

Pearson and Spearman correlation analyses showed that LCSs generally follow SRef trends, with correlations ranging from moderate to high, in line with similar studies [48,49].

However, Lin’s coefficient of concordance revealed that, while some sensors have reasonable concordances, others differ significantly, possibly due to calibration problems or sensitivity to uncontrolled environmental factors, as seen in the results of previous research [50,51].

Linear regression indicated that simple models do not fully capture the variability in measurements from these sensors, highlighting the need for more complex models and adjustments in factory calibration for applications requiring high accuracy. This is supported by comparable studies [52,53].

Differences in error metrics (MAE and RMSE) between sensors highlight which are more accurate and consistent, which is crucial for sensor selection in environmental monitoring, especially in contexts affecting public health or environmental compliance. This would support previous analyses [54].

The results of Student’s t and ANOVA tests suggest that there are no statistically significant differences between the means of sensor measurements and SRef for most cases. This indicates that, although the sensors may be comparable to the standard in general terms, more detailed analyses are needed to identify specific conditions under which the sensors may be less accurate. This would be in line with available knowledge [55].

Advances in LCS technology have expanded the opportunities for environmental monitoring, especially in the measurement of particulate matter such as PM2.5 and PM10 [56]. This study assessed the effectiveness of several inexpensive sensors and their factory calibration, compared to an SRef, using statistical analysis to examine their accuracy, reliability, and consistency. Finally, the results obtained allow us to consider the Sensirion SEN54 as the best factory alternative, to be assessed in future trials. However, the Nova SDS011 and the Sensirion SPS30 could be interesting alternatives to be evaluated with an adequate prior calibration.

In summary, this research on LCS compared with an SRef aimed to evaluate and validate their results and consider them as alternatives or as supportive systems to traditional monitoring networks, highlighting fundamental differences in terms of cost, accessibility, precision, and maintenance requirements, shedding light on the particular advantages of each in specific contexts. LCSs offer an economical and accessible alternative, ideal for noncritical applications, early anomaly alert, and environmental awareness and education initiatives [57]. Although their sensitivity to environmental variables such as humidity and temperature necessitates frequent calibrations, innovations in their technology and integration with IoT infrastructures are extending their practical applications. These advances allow LCS to effectively complement traditional monitoring networks, helping to mitigate their spatial and temporal limitations.

In contrast, traditional monitoring systems, while requiring a more considerable initial investment and specialized maintenance, are essential for operations that demand the highest precision and reliability. Their robustness and infrequency in the need for recalibration make them optimal for complying with stringent regulations and for detailed data management in critical contexts.

This discussion underscores the importance of selecting the appropriate monitoring system based on the specific needs of the project and operational conditions. Integrating both systems can not only optimize the coverage of environmental monitoring but also enhance the quality and precision of the data collected.

## 5. Conclusions

Advancements in LCS have facilitated their comparison with SRef, which is crucial for assessing their application in the collection of high-resolution spatial and temporal data. According to the hypothesis presented in this research, LCSs are useful in indoor environments of factories and laboratories, where humidity levels are controlled and fluctuations are limited by air conditioning, and where sources of particulate material are known and less varied than outdoors. This could contain and minimize the main limitations and sources of error of LCSs. It would offer multiple advantages to the managers of these environments, as LCSs allow the monitoring of multiple different spaces without interfering with operations, enabling the mitigation of workers’ exposure to particles, offering quick, economical monitoring of sufficient quality to generate real time alerts, as well as traceability of particulate material in workspaces, with data that can be reviewed by external audits or governmental occupational health inspections. Presenting a balance of the advantages versus limitations is more positive than in other monitoring applications, where LCSs could be a real alternative to traditional monitoring systems that are much more expensive, bulky, and complicated to manage and operate.

It was identified that among the existing LCSs in the market, starting from their factory precalibrations and subjected to this study, the Sensirion SEN54 is the most suitable for evaluation in more complex and real application studies. However, this research has also demonstrated that, to guarantee the accuracy and reliability of these devices, one cannot rely solely on factory precalibrations. It is necessary to perform local calibrations and validations to properly adjust the monitoring and ensure that the data obtained are relevant and useful for decision making.

Furthermore, it was observed that the exclusive use of the R^2^ coefficient is insufficient to capture all the variability and accuracy of the sensors. Therefore, it is essential to employ multiple statistical metrics, such as MAE, RMSE, and the Pearson and Spearman correlation coefficients, to achieve more precise and comprehensive evaluations, suggesting that reducing the use of statistical metrics in sensor evaluation to save resources could limit our ability to properly understand sensor performance, as each metric provides indispensable information for a comprehensive evaluation.

The need for regular calibration and adjustment to improve sensor performance is also highlighted, aligning with best practices in data analysis and minimizing problems from deviations and anomalies in monitoring due to equipment degradation.

For these reasons, we consider that the Nova SDS011 and Sensirion SPS30 devices could also be interesting for the development of a monitoring and control network for particulate matter in the air of indoor spaces with the proper adjustments and controls.

Furthermore, following the research, the use of a methodology and an environmental urn as described in the assay is validated. Due to its simplicity, low cost, and scalability for conducting simplified comparative LCS tests, which would facilitate the replicability of the tests by various research groups, contributing to the improvement of the comparative scientific study of these devices, their assessment and inclusion in regulations by regulatory bodies enhance and make more frequent the monitoring of indoor spaces, actively contributing to the health and quality in the workplace of the users of these monitored spaces.

For future research, we propose expanding the analysis by incorporating a greater number of low-cost system (LCS) models for particle monitoring. This would include an increase in the number of units evaluated per model, in order to conduct a more comprehensive comparative and a deeper analysis of the performance of these devices against governmental reference sensors. The inclusion of these variables will allow for a more effective evaluation of the reliability and accuracy of the LCS under controlled conditions.

Additionally, it is proposed to incorporate environmental fluctuations into the study, with a particular focus on humidity. According to the reviewed literature, humidity is the factor that can generate the most anomalies in LCS measurements. Considering this factor is crucial for providing a more complete analysis under real conditions and estimating the compatibility of the LCS with governmental monitoring networks.

It is advisable to extend the analyses over a longer period of time. This would facilitate the detection of anomalies in the functioning of the LCS and would allow for the training of automated systems that minimize required maintenance and ensure greater reliability of the data obtained.

By extending these studies and improving the calibration of the LCS, the potential misuse of these devices could be significantly reduced, especially in applications outside the research domain, such as in citizen science and personal monitoring of air quality. Educating users about the limitations and uncertainties of these sensors could significantly enhance the practical applications of LCSs.

Finally, the data from this study suggest that LCSs are particularly interesting to evaluate in other applications where it is necessary to cover extensive areas with limited resources, such as in rural areas or countries with fewer economic resources. In these contexts, LCS could contribute to establishing support monitoring networks alongside regulated networks, provided they are properly calibrated and reviewed in real applications.

## Figures and Tables

**Table 1:** Statistical values between LCS and SRef data.

Sensor	MAE ^1^	RMSE ^2^	*p*-Value	F-Value	CCLin o CCC ^3^
PM2.5 SDS011	124.941	251.471	0.293	1.128	0.712
PM2.5 SEN54	109.450	217.635	0.366	0.833	0.774
PM2.5 SEN0460	165.815	296.888	0.14	2.251	0.471
PM2.5 SPS30	158.372	292.608	0.071	3.405	0.534
PM10 SDS011	160.818	308.791	0.269	1.25	0.64
PM10 SEN54	143.353	279.182	0.257	1.316	0.689
PM10 SEN0460	187.392	318.932	0.253	1.335	0.526
PM10 SPS30	147.114	295.901	0.067	3.516	0.675

^1^ Mean absolute error; ^2^ Root mean squared error; ^3^ Concordance correlation of Lin or coefficient (CCLin o CCC).

## Data Availability

Data available on request due to restrictions. The data presented in this study are available on request from the corresponding author. The data are not publicly available due to privacy reasons.
